# Effect of Homogenization Method and Carvacrol Content on Microstructural and Physical Properties of Chitosan-Based Films

**DOI:** 10.3390/foods10010141

**Published:** 2021-01-12

**Authors:** Zoila Flores, Diego San-Martin, Tatiana Beldarraín-Iznaga, Javier Leiva-Vega, Ricardo Villalobos-Carvajal

**Affiliations:** 1 Facultad de Ciencias Tecnológicas, Universidad Nacional de Agricultura, Carretera a Dulce Nombre de Culmí, km 215 Barrio El Espino, Catacamas, Honduras; zflores@unag.edu.hn; 2 Food Engineering Department, Universidad del Bío-Bío, Av. Andrés Bello 720, P.O. Box 447, Chillán 3800708, Chile; diesanma@egresados.ubiobio.cl (D.S.-M.); tatybeldarrain@gmail.com (T.B.-I.); javier.leiva.vega@gmail.com (J.L.-V.)

**Keywords:** nanoemulsified film, carvacrol, chitosan, physical properties, microstuctural properties

## Abstract

The use of EOs nanoemulsion to develop active edible films offers a new way to modify transport properties and to release active compounds while improving mechanical resistance, transparency, and antioxidant and antimicrobial activity. The aim of this study was to study the influence of homogenization conditions and carvacrol content on the microstructure and physical properties of edible nanoemulsified chitosan films. Film-forming emulsions (FFE) were prepared with chitosan (1.5%), Tween 80 (0.5%), and carvacrol (0.25%, 0.5%, and 1.0%); two homogenization methods were used (rotor-stator and rotor-stator followed by high-pressure homogenization). Film internal and surface microstructure was characterized by scanning electron microscopy (SEM) and film physical properties, such as mechanical, optical, and water barrier, were evaluated. Results showed that the high-pressure homogenization method promoted a significant change on film microstructure, leading to improved properties. Carvacrol droplets were smaller and homogeneously distributed in the film when 0.5% (*v*/*v*) carvacrol was incorporated (1:1 Tween 80: carvacrol ratio). As a consequence, emulsified films obtained at high pressure were less opaque, had greater elongation, and had a lower permeability to water vapor than those obtained by the rotor-stator method. Therefore, high-pressure homogenization is a good method to obtain edible emulsified films with desirable properties for food preservation.

## 1. Introduction

Recently, edible films and coatings have received considerable attention in the field of food preservation given the promising results obtained and their potential use as a green alternative to plastic packaging films [1,2]. Edible films and coatings have been used to improve gas and moisture barriers, mechanical properties, sensory perceptions, convenience, and microbial protection [3], all of which enhance the quality and shelf life of food products. These films can be prepared from a variety of biopolymers, including proteins, polysaccharides, lipids, and their combinations [4,5]. Among the biodegradable polymers, chitosan has been widely used to produce edible films because it is nontoxic and biodegradable. It also has an excellent film-forming ability, a broad antimicrobial activity, selective permeability to gases (CO_2_ and O_2_), and is compatible with other substances, such as vitamins, minerals, and antimicrobial agents [6,7,8,9,10,11]. Unfortunately, chitosan coatings and films have poor water vapor barrier properties due to their hydrophilic nature [12]. This is an important drawback because effectively controlling moisture transfer is a desirable property for most foods.

Recent research has therefore focused its efforts on developing composite films [13]. The combination of polysaccharides and some hydrophobic compounds (fats and oils) could produce films or coatings with optimized characteristics, especially with respect to their moisture barrier properties [14]. The incorporation of essential oils (EOs) and their components, such as hydrophobic constituents, into chitosan films is an interesting way to enhance film functional properties [15,16,17]. They also have a great antimicrobial activity against a wide variety of bacteria, molds, and yeasts [18] and are considered as natural preservatives and generally recognized as safe (GRAS) for human consumption. Carvacrol is one of the major components of EOs in oregano, thyme, and marjoram, and it has been used for many generations as a food additive [19].

Composite films can be categorized as a bilayer or an emulsion. In a bilayer composite system, lipids generally form an additional layer over the polysaccharide or protein layer. In the emulsified system, lipids are dispersed into a hydrocolloid solution, which is then cast and dried to obtain an emulsified film [20]. The structural properties and stability of the film-forming emulsion (FFE) have a great impact on the final film microstructure, which greatly determines its properties [21,22]. Stability of the FFE depends on its particle size and distribution, rheological behavior, and *ζ*-potential of the dispersed lipid particles [23]. The size and distribution of the lipid particles in the FFE before and after the drying process affects the final emulsified film microstructure. Solvent evaporation during FFE drying induces destabilization phenomena such as flocculation, coalescence, and creaming due to lipid concentration. The produced destabilization mechanisms will depend on the physical state of the lipids, composition of the continuous phase, and drying conditions [24]. Therefore, to reduce this negative effect, it is fundamental to improve the initial emulsion stabilization factors (i.e., reduce particle size, increase viscosity of the continuous phase, or incorporate emulsifiers) [25]. A small lipid droplet size and high emulsion stability during the film-drying process gives rise to a homogeneous distribution of lipid particles in the film, which contributes to a more efficient control of water vapor transfer [26].

To disperse oil in the film-forming aqueous phase, emulsifiers are required along with an appropriate homogenization process. Emulsifiers adsorb at the oil-water interface during homogenization and reduce surface tension between the oil and water, thereby enhancing emulsification and increasing emulsion stability [27,28]. Emulsifier concentration is an important factor to consider in emulsion development with a smaller droplet size because of its large surface area. It has been reported that oil droplet size decreases in nanoemulsions when emulsifier concentration increases in both high-energy [29] and low-energy [30] homogenization methods.

Different homogenization methods can be used to produce emulsions with oil droplets that are as small as possible. Rotor-stator homogenizers are often used in the food industry and are able to reach particle sizes in the range of 1 µm. The droplet size can be further reduced to less than 1 µm by applying high-pressure homogenizers to the system to produce a uniform emulsion composition with a higher stability [4].

Some studies have shown that adding carvacrol to edible films decreases its water vapor permeability because its highly hydrophobic nature affects the hydrophilic/hydrophobic balance of the film. Carvacrol can also decrease the oxygen permeability of edible films by forming a more porous microstructure and modifying their mechanical properties as a result of the development of structural discontinuities that cause less flexibility and resistance to fracture [31,32,33]; however the relationship between the microstructure and physical properties of emulsified films needs to be further investigated. In our previous work [34], we observed that incorporating carvacrol and using different homogenization methods significantly affected the physicochemical properties of chitosan-based film-forming emulsions. The present study is a continuation of the previously mentioned research study. Thus, the aim of the present work was to study the influence of homogenization conditions and carvacrol content on the microstructure of chitosan-based films and their impact on an optical, water vapor barrier, and mechanical properties of these emulsified films.

## 2. Materials and Methods

### 2.1. Materials

Carvacrol (>98% purity, CAS Number 499-75-2), chitosan with 810 KDa molecular weight and deacetylation degree >75%, glycerol, and Tween 80 were obtained from Sigma-Aldrich (St. Louis, MI, USA). Glacial acetic acid and absolute ethanol were purchased from Merck S.A. (Santiago, Chile). Ultrapure water was obtained from Direct-Q^®^ 5 UV filtration system (Millipore, Molsheim, France).

### 2.2. Preparation of Chitosan Solutions and Film-Forming Emulsions

Chitosan solutions and film-forming emulsions were prepared according to the method described in a previous publication [34]. A 1.5% (*w*/*v*) chitosan stock solution was prepared by dissolving chitosan powder in a hydroalcoholic acid solution (1% acetic acid and 2.5% ethanol). To achieve complete chitosan dispersion, the solution was stirred for 6 h at room temperature. Impurities were removed by vacuum filtration with a microfiber filter. Ethanol was used to decrease the surface tension of film-forming emulsions as described by Kurek et al. [35] The chitosan mass was plasticized with 15% glycerol, and the chitosan solution was stirred for 30 min.

The film-forming emulsions were obtained by adding 0.5% (*v*/*v*) Tween 80 as an emulsifier and carvacrol at 0.25%, 0.5%, and 1.0% (*v*/*v*) to the chitosan solution and were homogenized by two methods. In method 1, emulsions were prepared with a rotor-stator homogenizer (Ultra Turrax^®^ T25 digital, IKA WERKE, Staufen, Germany) at 21,500 rpm for 5 min. In method 2, the coarse emulsions obtained by the rotor-stator system were subjected to a second homogenization process with a high-pressure homogenizer (model NG 12500, Stansted Fluid Power Ltd., Essex, UK) at 100 MPa. Finally, vacuum degassing was used to remove dissolved air bubbles in the emulsions. The film-forming emulsion compositions are displayed in Table 1. For each composition, three separate batches of emulsions were prepared using both homogenization methods.

### 2.3. Preparation of Emulsified Films

The films were obtained by the casting technique. Films with uniform thickness were achieved by using a volume of film-forming emulsion containing the same amount of total solids. This volume was poured into a petri dish, weighed on an analytical balance, and dried for 24 h at 30 °C in an oven under slight orbital agitation. The resulting films were removed from the petri dishes and stored in a desiccator containing silica before being characterized.

### 2.4. Film Thickness

Film thickness was measured with a digital micrometer (Electronic Outside Micrometer, Fowler High Precision, Inc., Newton, MA, USA). Measurements were made in five different positions on each film sample and in triplicate for each film formulation. Mean values were used to evaluate the mechanical and water vapor barrier properties of the films.

### 2.5. Microstructural Characterization

The film surface and cross-sectional microstructure were examined with a scanning electronic microscope (JEOL JSM LV-6380, Tokyo, Japan). Film samples were cut with a sharp scalpel (5 × 1 mm) to analyze the surface. Film cross-section analysis was carried out in a previously cryo-fractured film specimen by liquid nitrogen immersion and then mounted on copper stubs perpendicularly to their surface. After gold coating, images were captured using 15 kV accelerating voltage.

### 2.6. Film Opacity

Film opacity was determined according to the method described by Han and Floros [36]. Films were cut into rectangular pieces and placed in the cell of a UV/visible spectrophotometer (Genesys 20, Thermo Scientific™, Waltham, MA, USA) and sample absorbance was obtained at 600 nm. For each treatment, film opacity was measured in triplicate and calculated by Equation (1):

Opacity = A/T(1)

where A is absorbance at 600 nm and T is film thickness in mm. A low value in the final result indicates higher transparency.

### 2.7. Mechanical Properties

Mechanical properties were analyzed by the tensile test at room temperature using a TA-XT2 texture analyzer (Stable Micro Systems, Godalming, Surrey, UK) with a 25 kg load cell equipped with tensile grips according to the ASTM standard method D882 (ASTM, 2001). Films were cut into rectangular strips (10 cm × 2 cm) and equilibrated at 55% relative humidity (RH) for 72 h in a desiccator containing a saturated nitrate magnesium solution. Equilibrated film specimens were placed in film extension grips and measured with an initial grip separation and crosshead speed of 80 mm and 1 mm/s, respectively. The mechanical parameters, tensile strength (TS) and percentage elongation (E%) at break, were evaluated in eight samples from each film.

### 2.8. Water Vapor Permeability

Film water vapor permeability (WVP) was evaluated gravimetrically by a modified version of the ASTM standard method E96-95 [37]. Stainless steel permeability cups (Cat#13-338, Fisher Scientific, Atlanta, GA, USA) were used to determine WVP. Cells were filled with distilled water (7 mL), and a circular sample of film was fixed in the upper part of each cell with high vacuum silicone grease and a metal screw ring clamp to ensure cell tightness. Cells were then placed inside the desiccators containing a NaCl saturated solution and a fan to homogenize and maintain a 100-75% RH gradient across the film. The desiccator containing the cells was placed in an oven at 25 °C, and cells were weighed every 2 h over 24 h to ensure a steady weight loss [38]. The WVP was calculated by Equation (2).

(2) $$\mathrm{WVP}=\left(1.157\times 10^{5}\right){\mathrm{WVTR}}_{m}\times \frac{L}{\left(P_{w1}-P_{w2}\right)}$$

where WVTR is the water vapor transmission rate, g/(h × m^2^); L is the film thickness, m; Pw_1_ is the partial water vapor pressure on the film underside, Pa; Pw_2_ is the partial water pressure at the upper film surface, Pa.

### 2.9. Film Solubility

Samples (2 × 3 cm) from each film were dried at 110 °C for 24 h (initial dry weight) and then immersed in a beaker with 50 mL of distilled water in a bath with constant shaking for 6 h at 25 °C. Samples were filtered and dried at 110 °C for 24 h to determine final dry weight. Finally, film solubility was measured using Equation (3).

(3) $$Solubility\;\left(\%\right)=\left(\frac{W_{i}-W_{f}}{W_{i}}\right)\times 100$$

where *W_i_* and *W_f_* are the initial and final sample dry weights.

### 2.10. Statistical Analysis

Results were analyzed by one-way and two-way analysis of variance (ANOVA) with Statgraphics Centurion XVI software (Statistical Graphics Corp., Herdon, DC, USA) to determine significant differences among treatments. Significance testing was performed by Tukey′s test, and differences were statistically significant at *p* < 0.05.

## 3. Results and Discussion

### 3.1. Physical and Optical Properties

#### 3.1.1. Thickness

Film thickness is an important characteristic that needs to be evaluated because it can affect its mechanical, optical, and water vapor barrier properties; its functionality as packaging is therefore also affected [39,40]. According to Han and Krochta [41], film thickness is influenced by the solid content of the film-forming solution and processing parameter. In the present study, film thickness was not affected by the homogenization method and carvacrol content (Table 2). Its values varied between 0.011 ± 0.002 mm and 0.014 ± 0.002 mm, although no significant difference was observed. This result was expected because of the procedure established to obtain the films. The same concentration of total solids and a slight orbital agitation were used during the drying process to maintain uniform film thickness. Similar results have been obtained by Fernandez-Pan et al. [42], who found no differences in thickness between chitosan films prepared with carvacrol.

#### 3.1.2. Microstructural Characteristics

**Figure 1:**
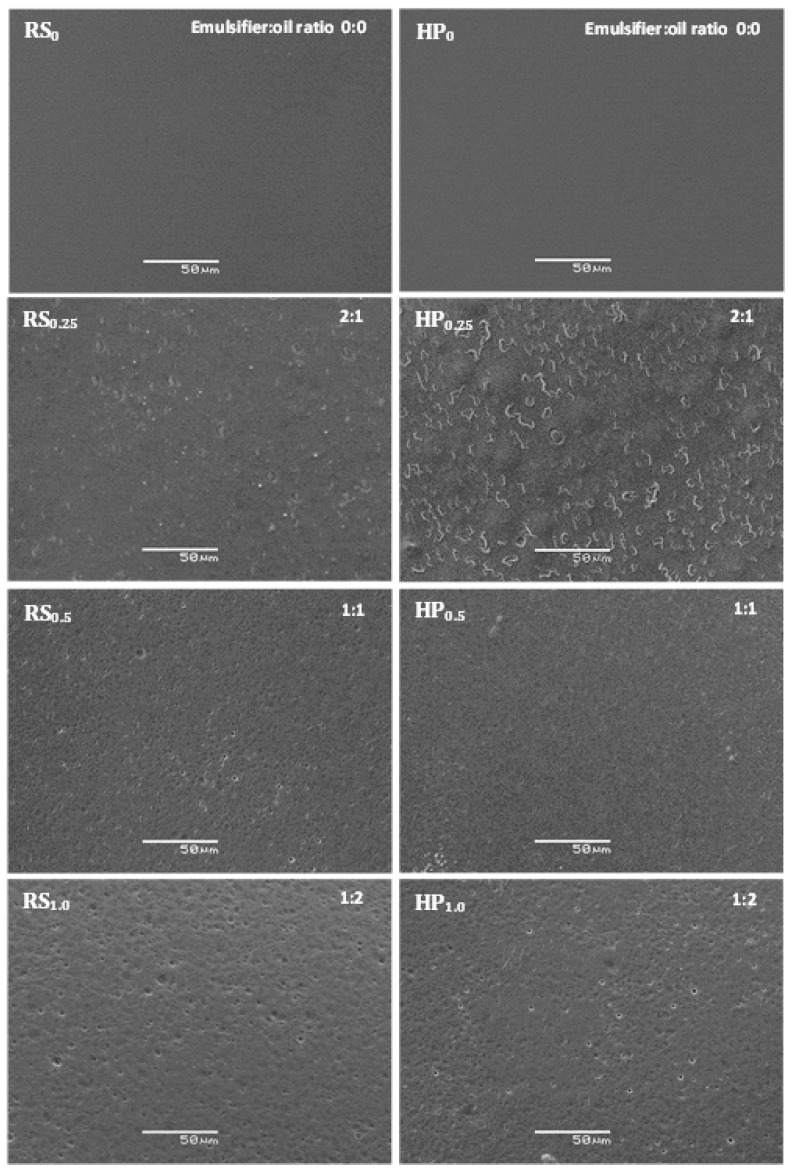
Scanning electron microscopy (SEM) micrographs of chitosan film surfaces with different carvacrol contents and homogenization methods. RS_0_, RS_0.5_, RS_1.0_, and RS_1.5_ correspond to films subjected to rotor-stator homogenization with 0, 0.5, 1.0, and 1.5% carvacrol. HP_0_, HP_0.5_, HP_1.0_, and HP_1.5_ correspond to films subjected to high-pressure homogenization with 0, 0.5, 1.0, and 1.5% carvacrol. Emulsifier:carvacrol ratios are shown in the figure.

Film microstructure was affected by the homogenization method, emulsion composition, and structural arrangement of the different components obtained at the end of the drying process. Film surface images by scanning electron microscopy (SEM) are shown in Figure 1. The surface of the control chitosan films, obtained by both homogenization methods, was smooth, uniform, and homogeneous without cracks. Similar results have been found in pure chitosan film prepared using a magnetic stirrer [4,31,32,43].

The addition of carvacrol resulted in a more heterogeneous surface structure with a distribution of oil droplets. The number of oil droplets on the surface increased with higher carvacrol concentrations. Films with 0.25% *v*/*v* carvacrol and homogenized by both methods had larger-sized structures with different surface morphologies compared to the other films. These films showed a 2:1 emulsifier:oil ratio. In this case, when the proportion of emulsifier is greater than oil, micellar aggregate formation can occur [44]. These structures could therefore correspond to micellar aggregates, which might be formed during the homogenization process and then have migrated to the surface during the drying process. However, a greater number of structures were observed on the film surface processed at high pressure. This phenomenon could be related to the lower viscosity exhibited by the emulsions obtained by this method compared to those processed by the rotor-stator system, which could facilitate the migration of the micellar aggregates toward the film surface. In this sense, several studies have shown that homogenization at high pressures can cause the reduction of the molecular weight of various biopolymers such as chitosan [45,46], alginate [47,48], and xanthan gum [49]. In these cases, modification of the molecular weight of biopolymers finally produced a reduction in the viscosity of the emulsions prepared with these biopolymers.

Although emulsions containing 0.5% carvacrol and homogenized by both methods had the smallest droplet sizes [34], it was possible to observe oil droplets and some pores on the surface of the films homogenized at high pressures (Figure 1). As previously noted, this behavior could be related to the lower viscosity of these emulsions. Under these conditions, the solvent evaporation rate during the drying process would also be faster, promoting oil droplet mobility toward the film surface. Essential oil droplet migration toward the film surface and pore formation due to oil volatilization during the drying process has also been observed by other authors [50,51,52]. At the highest carvacrol concentration (1.0% *v*/*v*), larger droplets were observed on the surface of films obtained using the rotor-stator system, and this is coherent with the largest droplet sizes found in the emulsions produced by this homogenization method [34].

The superficial microstructure of the films observed in the present study agree with the greater surface roughness detected by several authors when tea tree and ginger essential oils were incorporated into hydroxypropyl methylcellulose film [53,54] and thyme lemongrass and sage essential oils were incorporated into alginate films [50].

**Figure 2:**
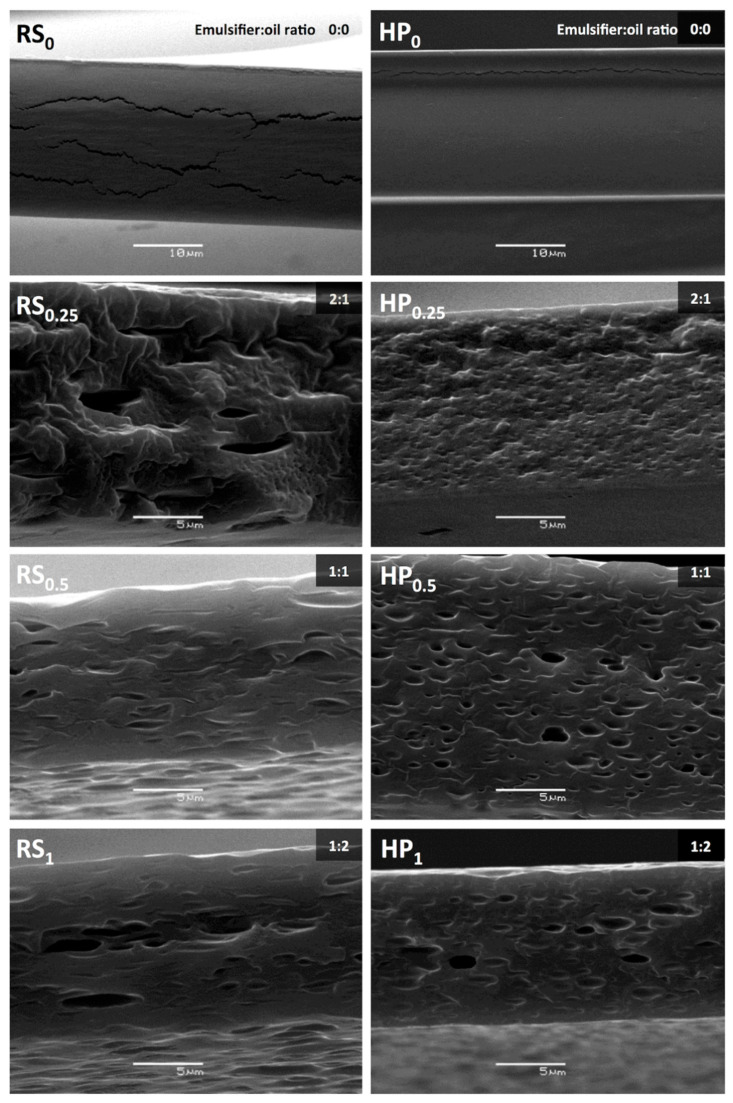
Scanning electron microscopy (SEM) micrographs of the chitosan film cross sections with different carvacrol content and homogenization methods. RS_0_, RS_0.5_, RS_1.0_, and RS_1.5_ correspond to films subjected to rotor-stator homogenization with 0, 0.5, 1.0, and 1.5% carvacrol. HP_0_, HP_0.5_, HP_1.0_, and HP_1.5_ correspond to films subjected to high-pressure homogenization with 0, 0.5, 1.0, and 1.5% carvacrol. Emulsifier:carvacrol ratios are shown in the figure.

The SEM cross section images of films prepared using both homogenization methods are shown in Figure 2. Chitosan control films showed some fractures parallel to the surface, which are possibly formed during the cryofracture process (Figure 2, RS_0_, HP_0_). Specifically, these films were more prone to fracture because a layered structure was formed during the drying process and generated a more compact and fragile matrix [55]. In spite of that, it is possible to observe a continuous and homogeneous structure, especially in films obtained at high pressure; this is possibly due to the reduced molecular weight of chitosan produced by the pressure applied in the homogenization process [45,46].

There were significant differences when carvacrol was incorporated in the internal microstructure of all the films. Emulsified films showed discontinuities associated with the presence of carvacrol droplets embedded in the continuous chitosan matrix, which were homogenously distributed across the film. Kurek et al. [35] described lipid droplets as having an ellipsoidal shape, being produced by the deformation forces caused by chitosan chain aggregation and orientation and matrix shrinkage during the drying stage. In general, the internal film microstructure depended on carvacrol droplet size and initial emulsion viscosity. When the emulsion droplet size is small (0.5% carvacrol), a more homogeneous internal microstructure is observed. A greater number of drops are in the near-surface area, especially in those films obtained from emulsions processed at high pressures, due to the lower initial emulsion viscosity.

However, when the emulsion droplet size was larger (0.25% and 1.0% carvacrol), emulsion destabilization during the drying process generated a more heterogeneous internal microstructure. Films with 0.25% carvacrol homogenized by both methods exhibited great differences in their internal structure. Films processed at high pressures had smaller homogeneously distributed carvacrol droplets and a surface layer formed by micellar aggregates composed of free emulsifier. According to a previous study, excess emulsifier forms micellar aggregates in an emulsion with a 2:1 Tween 80:carvacrol ratio (0.25% carvacrol) [34]. In contrast, films obtained by the rotor-stator system were more heterogeneous and had larger cavities. This could be related to the formation of micellar aggregates of the emulsifier that could not migrate to the surface because of its higher viscosity. In addition, small cavities are observed in some sections that could correspond to carvacrol drops. When the carvacrol content increased to 1.0% (1:2 Tween 80:carvacrol ratio), films obtained by both homogenization methods showed large droplets; because the emulsifier did not sufficiently cover all the droplets, this promoted its coalescence during the drying process. According to McClements [28], collision frequency between droplets in oil-in-water emulsions increases with a higher lipid content, which increases the probability of flocculation and the coalescence rate. Taking into account the structural characteristics observed in the film surface and cross section, more homogeneous films were produced by high-pressure homogenization, and a smaller carvacrol droplet size is distributed more homogeneously in the chitosan matrix. This occurred even in films with a high carvacrol content.

The effect of the incorporation of carvacrol on the internal and superficial microstructure of chitosan films, found in the present study, is in agreement with that described in a previous study. Kurek et al. [55] noted that the final microstructure of chitosan films is influenced by the structural arrangement achieved by chitosan and carvacrol in the film-forming emulsion, before and after drying. A low compatibility and miscibility between the components can produce a heterogeneous structure with a low stability during drying, promoting carvacrol droplet flocculation and subsequently their coalescence and creaming.

Finally, the creaming of the droplets, driven by solvent migration towards the evaporation surface, generated a more heterogeneous internal and superficial microstructure.

Bonilla et al. [4] have also described the influence of the homogenization method on the microstructure of chitosan-based films. They found results similar to those obtained in our study. The chitosan films incorporated with basil or thyme essential oil, by means of microfluidization at high pressures, showed a relatively more homogeneous microstructure than those films obtained by a rotor-stator homogenizer. The drop size reduction produced during high-pressure homogenization gave rise to increased interactions between the polymeric matrix and the essential oils, which led to a less cohesive chitosan matrix. In turn, it reduced the flocculation rate and the creaming of the droplets of carvacrol

#### 3.1.3. Mechanical Properties 

**Figure 3:**
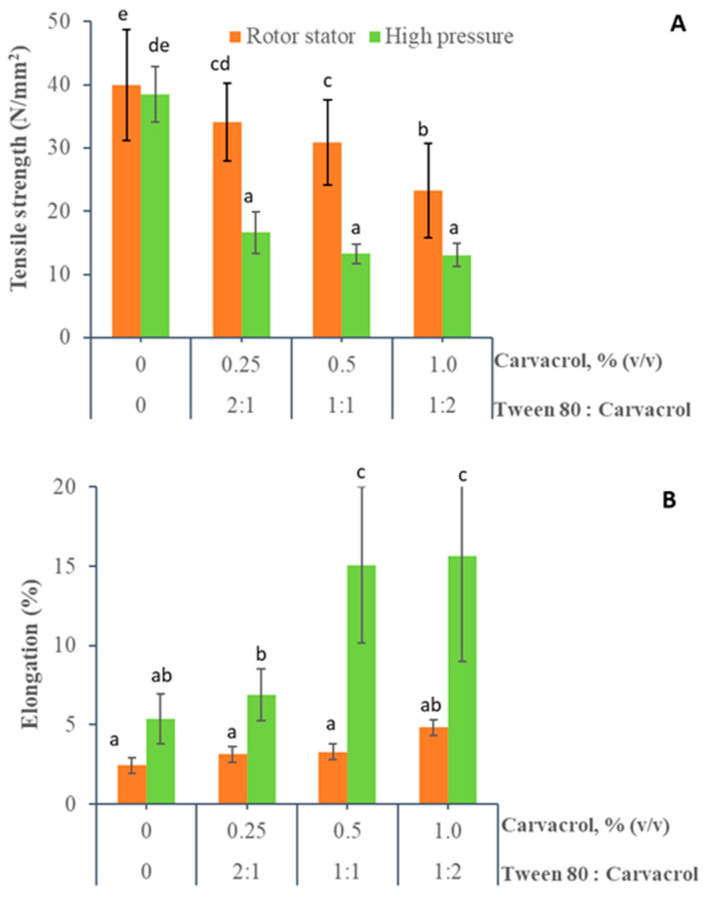
Effect of carvacrol incorporation and homogenization method on the mechanical properties of emulsified chitosan films. (**A**) Tensile strength and (**B**) Elongation. (a–e) Different letters indicate significant differences between treatments (*p* < 0.05).

Film mechanical properties are important characteristics for packaging materials because they determine their integrity and performance when applied and used in different situations [56]. The influence of carvacrol incorporation and the homogenization method on tensile strength (TS) and percentage elongation at break (E%) is shown in Figure 3. Control chitosan films obtained by rotor-stator and high-pressure homogenization showed the highest TS (39.95 ± 8.73 and 38.46 ± 4.85 N/mm^2^) and the lowest E% (2.45 ± 1.12% and 5.38 ± 1.74%), respectively.

The incorporation of carvacrol in the films significantly affected their mechanical properties. A significant decrease in TS was observed in the films obtained by rotor-stator (23.29 ± 7.49 N/mm^2^) and high pressure (13.05 ± 2.00 N/mm^2^) compared to the control chitosan film when the carvacrol content increased to 1.0%. In contrast, E% increased significantly to 4.84 ± 2.20% and 15.65 ± 7.18%, respectively. These effects were more pronounced in those films obtained from emulsions processed by high-pressure homogenization. According to the SEM images (Figure 2), film mechanical behavior would be influenced by an internal microstructure. When the carvacrol content increased in the films, a more heterogeneous internal structure with greater discontinuities and less cohesiveness was observed. These films also showed decreased TS and increased E%. However, changes in the mechanical properties of films obtained from emulsions processed at high pressures were more pronounced. These major changes would be associated with the formation of a more homogeneous internal microstructure than those obtained by the rotor-stator system (Figure 2) due to a smaller carvacrol drop size and the lower viscosity of these film-forming emulsions [34]. The cohesive strength of the film decreases when a larger quantity of small carvacrol droplets are homogeneously dispersed in the chitosan matrix; this contributes to the decrease in TS and improves film flexibility.

The reduction of TS and the increase in E% produced by incorporating different EOs or lipid compounds into chitosan films has also been described in various studies [4,57,58]. According to these authors, the presence of these lipid compounds produces a discontinuity of the chitosan matrix, which reduces the interactions between polymer chains and increases the plasticizing effect due to the increase of the oil droplet surface area in the polymer matrix.

#### 3.1.4. Opacity

**Figure 4:**
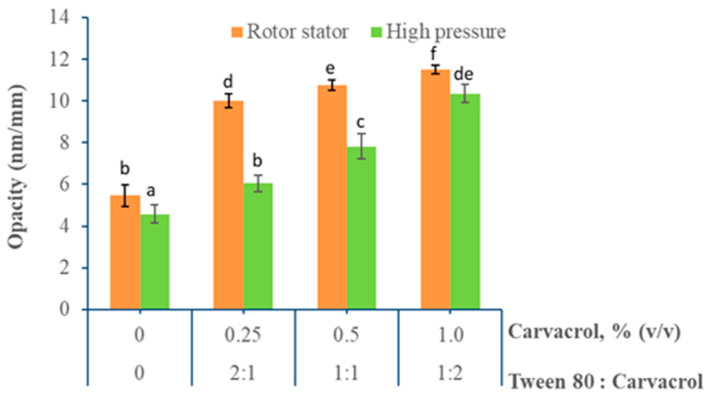
Opacity of emulsified films obtained by rotor-stator and high-pressure homogenization and different carvacrol contents. (a–f) Different letters indicate significant differences between treatments (*p* < 0.05).

Opacity is an important optical property that should be considered in the development of edible film because it has an important impact on appearance and determines the acceptability or rejection of coated products by consumers [53]. The effect of the homogenization method and carvacrol content on film opacity is shown in Figure 4.

The control film for both homogenization methods had the lowest opacity values, but these increased significantly (*p* < 0.05) when the carvacrol concentration increased. However, films obtained from emulsions processed at high pressures showed a lower increase in the opacity level compared to those emulsified by the rotor-stator system. It has been previously reported that the optical properties of films are affected by the developed internal and superficial microstructure because coalescence and creaming phenomena can occur during the film drying stage [22]. According to the SEM images, the formation of heterogeneous internal and superficial structures with increased carvacrol content is evident. Thus, the presence of these structures and their distribution in the film matrix decreases light transmission and increases its opacity. A similar trend has been reported by several authors when adding lipids to hydrocolloid films [43,59,60,61].

**Figure 5:**
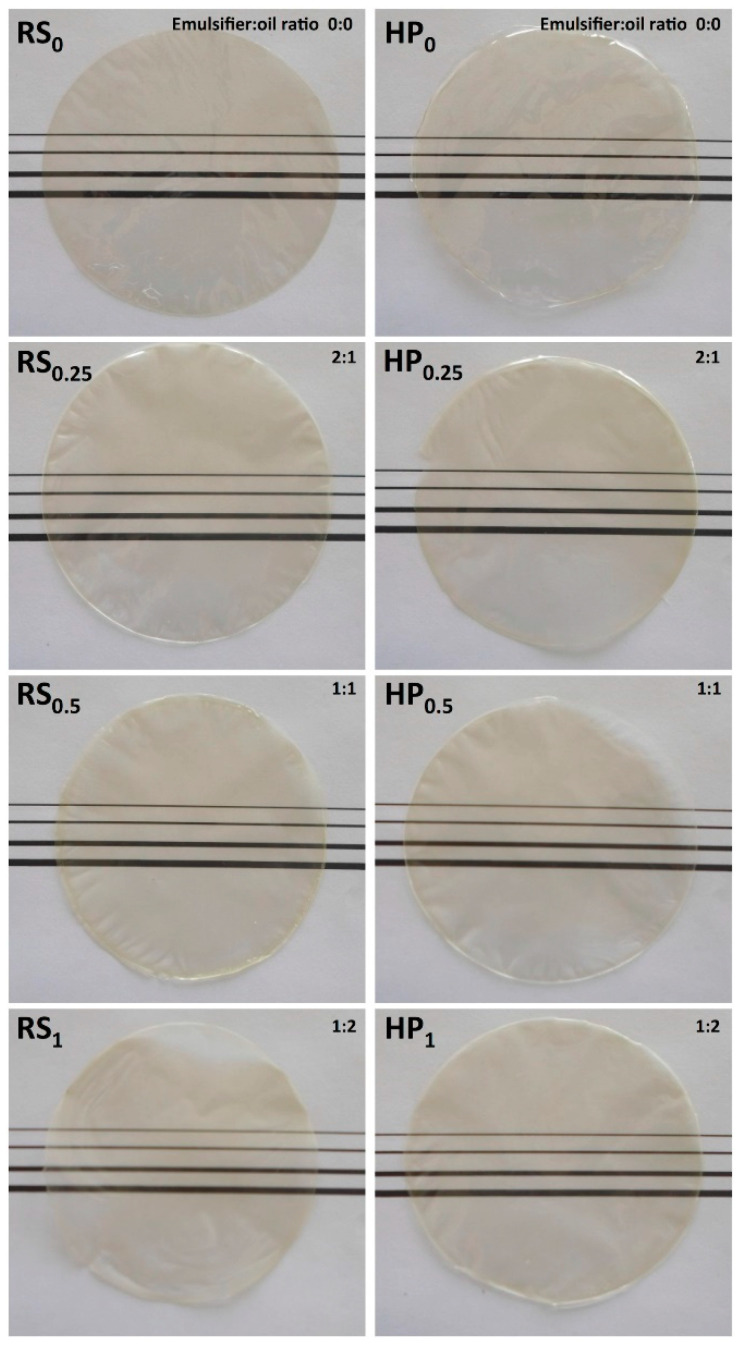
Photos of emulsified films obtained by rotor-stator and high-pressure homogenization and different carvacrol contents.

The lower opacity levels found in films emulsified by high pressures would be related to the more homogeneous microstructure attained by these films, which is due to the small carvacrol drops homogeneously distributed in the chitosan matrix. This same behavior can be confirmed when analyzing the film images shown in Figure 5. It can be observed that the control films, without carvacrol, are considered as transparent, but their opacity increased progressively when adding carvacrol. Films emulsified by high pressure also showed a lower opacity compared to those homogenized by the rotor-stator system.

#### 3.1.5. Water Solubility

**Figure 6:**
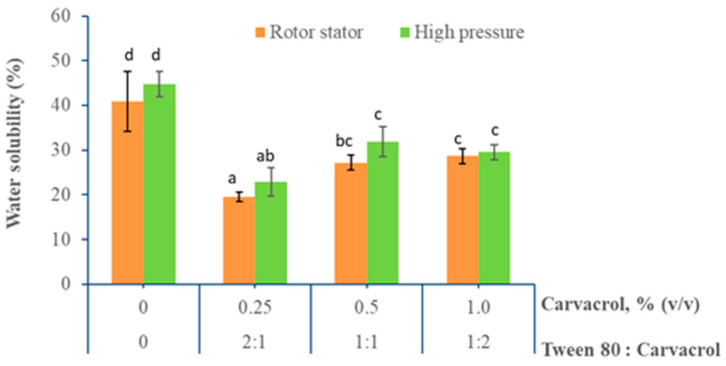
Effect of carvacrol content and homogenization method on water solubility of emulsified chitosan films. (a–d) Different letters indicate significant differences between treatments (*p* < 0.05).

Film water solubility is an important property of edible films to preserve food; it should be considered in their design because this property provides information about their water resistance as well as their behavior in aqueous environments. Figure 6 shows the effect of the carvacrol content and homogenization method on the water solubility of emulsified chitosan films.

The control chitosan films subjected to rotor-stator and high-pressure homogenization had a water solubility of 40.9 ± 6.7% and 44.7 ± 2.8%, respectively. These solubility levels could be associated with the presence of glycerol used as a plasticizer in the films, which has 3 hydroxyl groups [12], and with the protonated amino groups in chitosan that can interact with the water molecules to promote film solubility. This solubility was lower than the one reported by Lopez-Mata et al. [32], who studied chitosan film water solubility (54.6%).

This difference could be related to the greater film thickness (0.116 mm) and the higher chitosan concentration (2.0%) used in that study. The addition of carvacrol significantly decreased film water solubility compared to the control films in both homogenization methods. However, it was not possible to observe a trend to reduce film solubility when the carvacrol content increased. This result was unexpected because increasing the hydrophobic compound content in a hydrophilic matrix is expected to increase the hydrophobic nature of the matrix and hence reduce its water solubility. On the contrary, the lowest water solubility was obtained at the lowest carvacrol concentration (0.25%). A similar result has also been reported by Abdollahi et al. [62] when increasing rosemary oil concentration in chitosan films failed to decrease their solubility. This behavior can be related to the increase in the size of the carvacrol drops produced by coalescence during the drying process [63]. As a result of this increased drop size, the interfacial area is reduced, thus decreasing the amount of chitosan that interacts with the oil drop surface. This leads to an increase in the number of functional chitosan groups available to interact with the water molecules and favors film solubility.

#### 3.1.6. Water Vapor Permeability (WVP)

**Figure 7:**
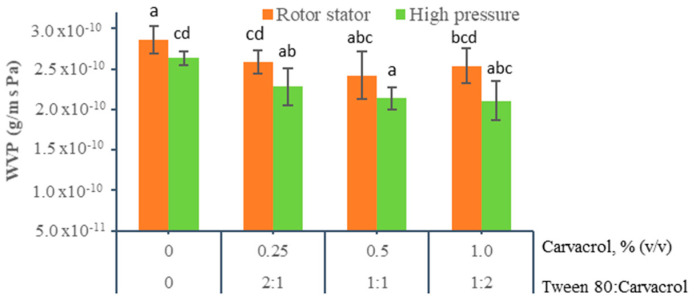
Effect of carvacrol content and homogenization method on water vapor permeability of emulsified chitosan films. (a–d) Different letters indicate significant differences between treatments (*p* < 0.05).

Water vapor permeability (WVP) is an important property that must be considered when developing food packaging. It should be as low as possible in order to delay moisture transfer between food and the surrounding environment and to achieve a longer shelf life [64]. The WVP of chitosan films containing carvacrol and subjected to different homogenization methods is shown in Figure 7.

The control chitosan films displayed the highest WVP values (2.86 × 10^−10^ ± 2.0 × 10^−11^ − 2.64 × 10^−10^ ± 3.01 × 10^−11^ g/(Pa × s × m)) and were not significantly affected by the homogenization methods. These results agree with those previously reported by Vargas et al. [46], who pointed out that the homogenization methods (rotor-stator and microfluidization) used to prepare chitosan films did not affect their WVP. The high WVP values shown by these films may be related to the hydrophilic nature of the chitosan matrix because reducing pH (4.5) is necessary for chitosan solubilization. At this pH level, chitosan amino groups are protonated and more reactive with the water molecules. In addition, the presence of glycerol in the chitosan matrix, which is used as a plasticizer, contributes to greater interaction with water molecules, favors its transfer through the film, and increases its WVP. The emulsified films by the rotor-stator system showed lower permeability values than those found by López-Mata et al. [32] in emulsified films using the same homogenization method. This low permeability could be explained by the higher molecular weight of the chitosan, the higher speed and time of homogenization and the lower amount of glycerol used.

The addition, carvacrol decreased the WVP in the films compared to the control films. However, this decrease (2.11 × 10^−10^ ± 1.70 × 10^−11^ and 2.29 × 10^−10^ ± 1.7 × 10^−11^ g/(Pa × s × m)was significant only in those films with 0.5% and 1.0% carvacrol and homogenized at high pressures. Films emulsified at high pressure, independently of the carvacrol content, exhibited lower WVPs than those homogenized by the rotor-stator system; however, these differences were not significant.

The lower permeability values found in films emulsified by high pressure could be related to the internal and superficial microstructure obtained at the end of the drying period. According to Figure 1 and Figure 2, the films with 0.5% and 1.0% carvacrol produced smaller-sized drops that were homogeneously distributed in the matrix and formed a superficial carvacrol layer. The discontinuities generated by the small carvacrol drops dispersed in the matrix therefore generated a more tortuous path for water molecule movement and decreased their diffusion through the film. Some authors point out that this tortuosity is greater when the oil phase ratio increases or when the oil particle size is reduced [65,66,67]. The formed carvacrol surface layer also acts as a barrier to increase its resistance to water vapor.

## 4. Conclusions

Carvacrol incorporation and the homogenization method being used had a significant impact on the microstructure and physical properties of the emulsified films. 

The emulsified film microstructure was determined by the destabilization phenomena experimented by the emulsion during its drying process. A more homogeneous microstructure was obtained when using the 1:1 Tween 80:carvacrol ratio and the high-pressure homogenization method. Under these conditions, greater emulsion resistance to destabilization was achieved during drying, and there was a more homogeneous distribution of the carvacrol in the chitosan matrix.

Carvacrol incorporation caused a significant impact on the mechanical and optical properties of the emulsified films. Significantly reduced tensile strength, increased elongation percentage, and increased opacity were promoted when carvacrol was added. This effect was more pronounced in high-pressure homogenized films because their internal and surface microstructure was more homogeneous.

The water solubility of the chitosan films and their water vapor permeability decreased when carvacrol was added. Lower solubility was related to the greater carvacrol droplet size in the chitosan matrix caused by the coalescence and formation of a surface layer during the drying stage. At the same time, high-pressure emulsified films exhibited lower water vapor permeability because of the homogeneous distribution of carvacrol droplets in the chitosan matrix and the formation of a surface layer, which generated a more tortuous path for water molecule movement.

In conclusion, the physical properties of the emulsified films depended on the microstructure developed during the drying stage. High-pressure homogenization is a good method to obtain edible emulsified films with desirable properties for food preservation.

## Figures and Tables

**Table 1:** Composition (100 mL) of film-forming emulsions.

Chitosan (g)	Glycerol (mL)	Tween 80 (mL)	Carvacrol (mL)	Emulsifier: Oil Ratio
1.5	0.225	0.0	0.0	0:0
1.5	0.225	0.5	0.25	2:1
1.5	0.225	0.5	0.5	1:1
1.5	0.225	0.5	1.0	1:2

**Table 2:** Thickness of chitosan films with different carvacrol content and homogenization methods.

Carvacrol (% *v*/*v*)	Thickness (mm)	
	Rotor-Stator	High-Pressure
0.0	0.013 ± 0.001 ^a,x^	0.012 ± 0.002 ^a,x^
0.25	0.012 ± 0.001 ^a,x^	0.014 ± 0.002 ^a,x^
0.5	0.011 ± 0.002 ^a,x^	0.013 ± 0.004 ^a,x^
1.0	0.012 ± 0.004 ^a,x^	0.012 ± 0.002 ^a,x^

^a^ Superscript within the same column indicates significant differences between formulations. ^x^ Superscript within the same row indicates significant differences between homogenization methods.

## Data Availability

Publicly available datasets were analyzed in this study. This data can be found here: https://drive.google.com/file/d/1Y5ORtRuElBTviNrwxQDoCq6W5CnMyi5H/view?usp=sharing.
